# Pathogenesis of chikungunya arthritis: A scoping review

**DOI:** 10.1371/journal.pgph.0005955

**Published:** 2026-03-03

**Authors:** Mario Rankin, Somasundram Pillay, Jasmine Ramiah, Leonard Marais

**Affiliations:** 1 Department of Orthopaedics, University of KwaZulu-Natal, Durban, South Africa; 2 Department of Internal Medicine, University of KwaZulu-Natal, Durban, South Africa; 3 Department of Clinical Haematology, University of KwaZulu-Natal, Durban, South Africa; 4 Department of Orthopaedics, School of Clinical Medicine, UKZN, Durban, South Africa; PLOS: Public Library of Science, UNITED STATES OF AMERICA

## Abstract

Chikungunya virus (CHIKV) is a mosquito borne alphavirus that causes chikungunya disease, which is characterized by debilitating arthritis. While the acute phase is marked by fever and joint pain, many patients develop chronic arthritis that persists for years. The pathogenesis of chikungunya arthritis (CA) is not fully understood, prompting us to perform an updated review of the literature to describe the mechanisms contributing to this condition. A comprehensive search was conducted on numerous electronic databases for all studies relating to pathogenesis of CA. The search strategy included the key word search “pathogenesis”, “chikungunya”, “arthritis”. Two reviewers independently determined eligibility, rated study quality, and extracted data. The methodology followed the Preferred Reporting Items for Systematic reviews and Meta-Analyses extension for Scoping Reviews. The data from included articles were charted and a qualitative thematic analysis formulated. A total of 623 records were identified from the electronic database search. This was narrowed to 63 records which were eligible after excluding by title and then by abstract. Following the surveying of 58 available full texts, 35 articles were eligible for inclusion, from which data was extracted. Key mechanisms identified for chronic CA included host risk factors, viral component persistence in joints, a robust pro-inflammatory cytokine response, the resulting joint destruction from pannus formation and the activation of matrix metalloproteases (MMPs) and osteoclasts. This scoping review extrapolates factors that contribute to the pathogenesis for developing chronic CA. Namely host factors and the ability of CHIKV to establish persistence within synovial fibroblasts. The inflammatory mediators and signalling pathways triggered by the host fibroblast synoviocytes, the secretion of MMPs, and promoting of osteoclastogenesis, contribute to ongoing inflammatory immune response and joint destruction. Thus, this review synthesizes evidence for a multifactorial pathogenesis of chronic CA, paralleling mechanisms of rheumatoid arthritis and highlighting targets for potential therapeutic intervention.

## Introduction

Chikungunya virus (CHIKV) is an arthropod-borne alphavirus transmitted primarily by the *Aedes* species of mosquitoes. It is characterized by fever, skin rashes and often accompanied by debilitating joint pain and inflammation [1]. Hence the name “Chikungunya” taken from Makonde language (a native Tanzanian language) meaning “to be contorted” or “that which bends” [1–3]. While the acute phase of the disease is self-limiting, a large proportion of patients develop chronic arthritis, which can persist for months to years. This chronic phase of the disease is known as chronic chikungunya arthritis (CA) which often leads to significant morbidity and poor quality of life [4]. Amaral et al. noted that more than 40% of patients develop prolonged arthritic disease lasting more than 3 months following the acute phase of illness [5].

Geographical areas with reported cases of disease transmissions within the last 5 years include Central and South America, Central Africa, South-East Asia and Malaysian islands[6]. It is reported that approximately 80 000 cases have been documented worldwide in 2025 alone, with 14 CHIKV related deaths in the same year[7].

The pathogenesis of CA is poorly understood and multifactorial, involving various host factors, viral persistence, various inflammatory triggers and signalling pathways from the host immune response, and eventually followed by cartilage and bone destruction within synovial joints. It has been suggested that the pathogenesis may be closely related to rheumatic manifestations [2,8].

CHIKV pathogenesis begins at the site of mosquito inoculation, where the virus initially infects dermal fibroblasts and macrophages as well as other immune cells withing the skin. Early viral replication in the skin activates pattern-recognition receptors, particularly Toll-like receptors (TLR-3, TLR-7, and TLR-8), stimulating the production of type I interferons (IFN-α/β) and pro-inflammatory cytokines. The virus then disseminates through lymphatic vessels to regional lymph nodes and subsequently into the bloodstream, leading to viremia and systemic spread. CHIKV displays marked tropism for musculoskeletal tissues, including synovial fibroblasts, skeletal muscle satellite cells, and macrophages within joint tissues. Viral replication in these tissues, coupled with the host’s innate and adaptive immune responses, establishes the foundation for acute inflammation and, in many individuals, progression to chronic arthritis despite declining viral loads [9,10].

Ultimately, the joint destruction is not curable and thus in many cases may require joint replacement surgery. It is thus relevant for orthopaedic practitioners to understand this condition and its pathogenesis. There may be ongoing inflammatory processes involving the synovium, and thus some symptoms are likely to persist even following arthroplasty surgery [11].

Given the heterogeneity of available studies, which include clinical cohorts, in-vitro mechanistic research, autopsy findings, and narrative reviews, this scoping review was chosen to map the existing evidence, clarify areas of consensus, and identify key gaps for future research. This approach aligns with the objectives of PRISMA-ScR, emphasizing breadth and thematic synthesis over quantitative comparison.

This scoping review aims to synthesize a synopsis of the current understanding of the pathogenesis of chikungunya arthritis based on available literature.

## Methods

A comprehensive search was conducted in the following electronic databases:

PubMed MEDLINE, Scopus, Web of Science and Google Scholar from their inception up to and including October 2024 for all studies that discussed the pathogenesis of CA. The search strategy included the key word search “pathogenesis”, “chikungunya”, “arthritis”. The search was refined to only include literature in the English language, and studies of the human species.

The methodology followed the Preferred Reporting Items for Systematic reviews and Meta-Analyses extension for Scoping Reviews (PRISMA-ScR). Two reviewers independently determined study eligibility and thereafter the main author extracted data. Although we noted study quality, it did not exclude studies. The two reviewers screened the search results of all identified titles and came to a consensus of eligibility by title. From these titles the two reviewers screened abstracts of eligible records and came to a consensus of choice for inclusion by abstract. The single reviewer (main author) then reviewed all full texts of the eligible abstracts to identify appropriateness for inclusion and data extraction.

Journal articles were the only study type considered to be part of the inclusion criteria. Included sources were primary research or reviews that discussed information on mechanisms of CHIKV arthritis. We excluded animal studies, papers on systemic (non-arthritis) pathogenesis, vaccine or therapeutic trials, and clinical reports that lacked pathogenesis data. The data from included articles were charted and grouped into common themes of pathogenesis factors, which form the basis of our results narrative.

Although animal studies were excluded from formal evidence synthesis, mechanistic insights from murine models were occasionally referenced only where primary human studies cited them directly to contextualize immunological pathways.

## Results

A total of 623 records were identified from the chosen electronic databases, of which 494 records remained after duplicates were removed. From these, following consensus from the two reviewers, the number of records selected by title were 175, and thereafter 63 of those records were found to be eligible after analysing the abstracts. Following the surveying of the 58 available full texts (5 full texts were not accessible), 35 articles remained eligible, from which data was extracted.

**Fig 1:**
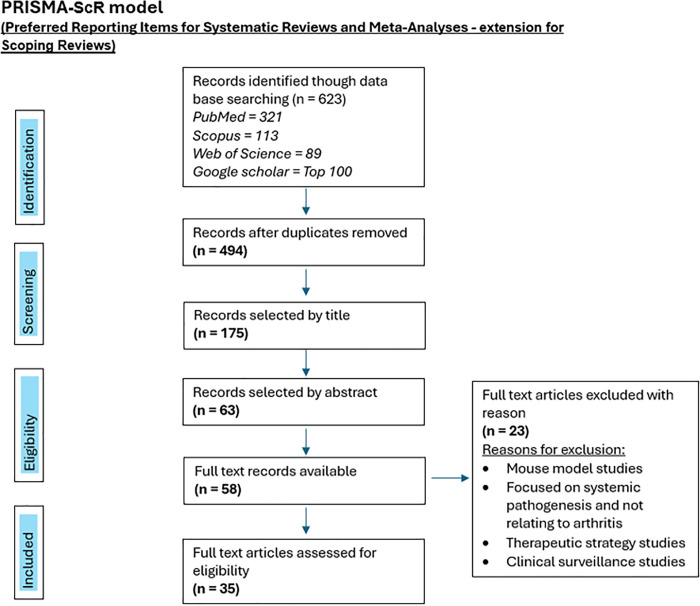
PRISMA flow diagram showing a selection of included studies.

It is important to note that despite the initial search being refined to human studies, there were a number of studies which still included mouse models within their methodology, and these were excluded during full text surveillance (Fig 1).

Most publications analysed emanated from Brazil (n = 9). This was closely followed by France (n = 7). This appeared to be due to the work done following the CHIKV outbreaks in La Reunion Island in 2005 and 2006. All eligible sources were published between the years 2010 and 2024.

Included studies (n = 35) comprised:

- Clinical cohort or longitudinal studies (n = 14)
- Cross-sectional or case-controlled immunological studies (n = 9)
- In-vitro studies using human-derived fibroblasts, macrophages, or synovial cells (n = 7)
- Narrative or mechanistic reviews with substantive immunopathogenic detail (n = 5)

A summary of included study types and primary pathogenic focus is presented in Table 1.

**Table 1:** Included studies in the scoping review of Chikungunya arthritis pathogenesis.

Reference	Study type/ samples	Main pathogenic focus
Hoarau et al., 2010	Human longitudinal cohort; synovial tissue, serum	Persistent inflammation; viral RNA/proteins in synovial macrophages
Burt et al., 2017	Narrative review	Clinical and immunopathogenic overview of CHIKV
Schwartz & Albert, 2010	Narrative review	Early infection, innate immunity, dissemination
Couderc & Lecuit, 2015	Narrative review	Tropism, immune evasion, chronic disease
Amaral et al., 2019	Narrative review	Clinical features and methotrexate therapy
Amaral et al., 2023	Narrative review	Links between CHIKV arthritis and RA
Silveira-Freitas et al., 2024	Review	Immunopathology of persistent arthralgia
Ali Ou Alla & Combe, 2011	Review	Post-CHIKV arthritis features
Dupuis-Maguiraga et al., 2012	Clinical cohort; serum	Acute-to-chronic inflammatory markers
Chow et al., 2011	Longitudinal cohort; serum	IL-6, GM-CSF and persistent arthralgia
Chang et al., 2018	Prospective cohort; serum	Cytokine profiles predicting chronic disease
Ninla-Aesong et al., 2019	5-year follow-up cohort	Cytokines as biomarkers
Chaaithanya et al., 2011	Cross-sectional cohort	Proinflammatory cytokines in chronic arthropathy
Chaaithanya et al., 2014	Clinical imaging study	Persistent erosive arthritis
Ozden et al., 2007	In-vitro human muscle cells	Musculoskeletal tropism
Her et al., 2010	In-vitro human monocytes	Innate immune activation
Fros et al., 2010	In-vitro human cells	Interferon pathway inhibition
Lum et al., 2015	Review	Cellular mechanisms of pathogenesis
Ng, 2017	Review	Immunopathology from human and animal data
Valdés-López et al., 2022	In-vitro human monocytes	Vitamin D modulation of inflammation
Kulkarni et al., 2017	Clinical cohort	Regulatory T cells and IL-10
Liu et al., 2022	Experimental human data	Role of IL-17
Banerjee & Saha, 2018	Clinical cohort	Oxidative stress markers
Atella et al., 2023	Review	Macrophage role in alphavirus arthritis
Chen et al., 2015	Review	Bone pathology in alphavirus arthritis
Avila-Trejo et al., 2023	Review	Mechanisms of bone damage
Phuklia et al., 2013	In-vitro human FLS	Osteoclastogenesis
Jaffar-Bandjee & Gasque, 2012	Clinical review	Chronic arthritis pathology
Bouquillard & Combe, 2009	Clinical case series	RA following CHIKV
Thon-Hon et al., 2012	In-vitro human fibroblasts	Fibroblast immune responses
Poo et al., 2014	Human-referenced mechanistic study	CCR2/MCP-1 axis
Gardner et al., 2010	Contextual animal study	Macrophage-driven arthritis
Chen et al., 2015 (Bindarit)	Experimental translational study	MCP-1 inhibition and bone loss
Schilte et al., 2010	Human-referenced experimental study	Type I IFN control
Priya et al., 2014	In-vitro human cells	TLR7/8 viral sensing

From the 35 eligible records, the following themes emerged: Pre-existing host risk factors, the ability for the chikungunya virus to establish persistence within the human host, the varying mechanisms of inflammatory triggers and signalling pathways and the ability to cause bone and joint destruction.

**Fig 2:**
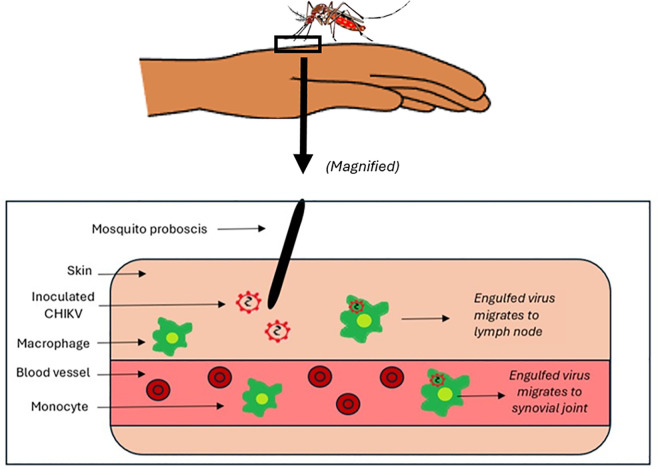
Illustration of the inoculation of the CHIK virus by the mosquito into the skin tissue of the human host, subsequently followed by some of the virus being engulfed by macrophages.

The immune response begins soon after inoculation through a bite from an infected mosquito Fig 2. CHIKV then disseminates in the individual through the circulation. Skin, lymph nodes, spleen, liver, muscle and fibroblasts within synovial joints are sites of primary replication. At this acute phase a robust antiviral immune response has already been activated in the target tissues which is characterized by an infiltration of predominantly macrophages, but also includes a response from CD4+, CD8+, B cells, Natural Killer (NK) cells and neutrophils [12]. As with most alphaviruses, CHIKV displays marked tropism for musculoskeletal tissues, including synovial fibroblasts, skeletal muscle satellite cells, and macrophages within joint tissues. Viral replication in these tissues, coupled with the host’s innate and adaptive immune responses, establishes the foundation for acute inflammation and, in many individuals, progression to chronic arthritis despite declining viral loads [12].

### 1. Host factors

#### Genetic predisposition.

Host genetic factors play a crucial role in determining the susceptibility to, and severity of CA. Several studies have identified genetic polymorphisms associated with an increased risk of developing chronic arthritis following CHIKV infection. For instance, polymorphisms in the human leukocyte antigen (HLA) genes, namely HLA-DRB1*04-HLA-DQB1*03 haplotypes have been linked to a higher risk of chronic CA [13,14]. These HLA alleles are involved in antigen presentation and may influence the adaptive immune response to CHIKV, leading to prolonged inflammation and joint damage. Polymorphisms of Toll Like Receptors (TLR) in particular TLR-7 and TLR-8 expressed on dendritic cells and monocytes, have been implicated in altered viral sensing and cytokine production, contributing to more severe or persistent inflammation [3].

#### Age and gender.

Age and sex are also important host factors influencing the pathogenesis of chikungunya arthritis [15]. Women are more frequently affected by chronic CA than men, suggesting a potential role of sex hormones in modulating the immune response to CHIKV [3,16,17]. Oestrogen, for example, has been shown to enhance the production of pro-inflammatory cytokines, which may contribute to the persistence of joint inflammation [18]. Age above 45 years was cited in a number of sources as an independent risk factor for chronic CA likely owing to the fact of having pre-existing joint disorders (e.g., osteoarthritis) in patients with advanced age [16,17,19], as well as higher viral loads having been detected in elderly patients [20,21]. It is also increasingly evident that the elderly are particularly susceptible to severe disease from viral infection likely due to the dysregulation of their immune system [21].

#### Comorbidities.

Comorbidities such as diabetes, hypertension, and obesity have been associated with an increased risk of developing chronic chikungunya arthritis [17,21]. These conditions are characterized by chronic low-grade inflammation, which may exacerbate the inflammatory response to CHIKV infection and contribute to the development of chronic arthritis. An interesting in-vitro study, by Valdez-Lopez et al., noted the ability of Vitamin D3 to modulate pro-inflammatory cytokines, without affecting viral clearance [22]. Thus, it was proposed that Vitamin D deficiency may be a predisposing host factor for CHIKV arthritis.

### 2. Viral persistence

#### Mechanisms of viral persistence.

Viral persistence is believed to be a key factor in the pathogenesis of CA. CHIKV has been detected in joint tissues, synovial fluid, and macrophages of patients with chronic arthritis, suggesting that viral elements can establish a persistent inflammatory response in these tissues [21]. Human studies, particularly the longitudinal cohort by Hoarau et al., have demonstrated the presence of CHIKV RNA, viral proteins and other persistent activation signatures within synovial macrophages, fibroblast-like synoviocytes, and less frequently in muscle tissue. There is currently no definitive human evidence of fully replication-competent virus during the chronic phase. Rather, persistence appears to reflect long-lived intracellular CHIKV RNA remnants, non-productive viral proteins, and/or persistent immunogenic debris [21,23,24].

Chronic viral antigen exposure can theoretically induce exhaustion phenotypes in T-cells. T-cell exhaustion following a robust immune antiviral response was described by Mueller et al., and this is thought to contribute to subsequent viral persistence [25]. This however was described in lymphocytic choriomeningitis virus in mice and direct evidence in human CHIKV arthritis is limited.

#### Role of immune evasion.

CHIKV utilizes a number of strategies to evade the host immune response and thus allowing viral persistence in the joints. The virus can inhibit the production of type I interferons (IFNs), which are involved in the antiviral immune response [15]. Fros et al. described CHIKV non-structural protein nsP2 has been shown to interfere with the IFN signalling pathway, preventing the expression of IFN-stimulated genes (ISGs) that are essential for viral clearance [26]. Hoarau et al. showed the presence of Type1 IFN in the synovial tissue of patients with chronic CHIKV infection in their longitudinal cohort study [21]. In addition, CHIKV can evade immune recognition by down regulating the expression of major histocompatibility complex (MHC) class I molecules of infected cells, reducing their ability to present viral antigens to cytotoxic T-cells [27].

CHIKV can also evade host immune response through modulation of apoptotic mechanisms. CHIKV is known to induce apoptosis and that virus-laden apoptotic remnants can be engulfed by macrophages, allowing the virus to spread while avoiding immediate immune detection [28].

#### Impact of viral persistence on joint pathology.

The persistence of CHIKV in joint tissues contributes to the chronic inflammation and tissue damage observed in chikungunya arthritis. The presence of persistent viral elements leads to the continuous activation of the immune system, resulting in the sustained production of pro-inflammatory cytokines and chemokines. This chronic inflammatory environment promotes the infiltration of immune cells into the joints, leading to synovitis, cartilage degradation, and bone erosion [15,29]. Furthermore, the presence of viral RNA and proteins in joint tissues can directly induce the production of matrix metalloproteinases (MMPs) and other enzymes that degrade extracellular matrix components, contributing to joint destruction [12,30]. These structural changes contribute to long-term disability and occasionally require surgical interventions.

### 3. Inflammatory triggers and signalling pathways

Multiple sources focused on various notable inflammatory triggers. These include a wide range of immune cellular responses, cytokine response and chemokine response. We will focus on the most commonly mentioned proinflammatory mediators contributing to the immune signatures in chikungunya arthritis. Note that, despite excluding animal models from our search process, many of the human studies discuss and refer to data derived from mouse models.

Several signalling pathways are involved in the pathogenesis of chikungunya arthritis, including the Janus kinase-signal transducer and activator of transcription (JAK-STAT) pathway, cytokines and chemokines, monocytes/macrophages and Natural Killer (NK) cells. These pathways are activated in response to CHIKV infection and play a critical role in regulating the pro-inflammatory response [21].

The Type 1 IFN immune pathway is a crucial anti-viral response which is well documented in the literature in the context of CHIKV [29,31]. This pathway activation is initiated in part, by macrophages which secrete cytokines such as IL-6, and NK cells which predominantly secrete IFN- γ. These cytokines are known to be elevated in chikungunya arthritis [15,20,31,32]. The pathway ultimately leads to the production of Type 1 IFNs which signal to subsequently amplify the anti-viral cascade mediated through the Janus Kinases and signal transducers and activators of transcription (JAK-STAT) pathway. Activation of the JAK-STAT pathway leads to the transcription of genes involved in inflammation and immune regulation [31,33]. Dysregulation of the JAK-STAT pathway has been implicated in the pathogenesis of other chronic inflammatory diseases, including rheumatoid arthritis, and may also play a role in chikungunya arthritis [31].

#### Cytokine storm.

The pre-arthritic acute phase of CHIKV infection is characterized by a massive release of pro-inflammatory cytokines and chemokines. The acute and chronic phases exhibit overlapping but distinct cytokine profiles. In the acute phase we note the presence of cytokines IL-1β, IL-6, TNF-α, IL-8, IFN-γ, IL-17 and early chemokines (MCP-1/CCL2, CXCL9, CXCL10). However, during the chronic phase of persistent arthritis we note sustained elevation of IL-6, increased granulocyte macrophage colony stimulating factor (GM-CSF), persistent IFN-γ production, and reduced anti-inflammatory signals such as IL-10 in some cohorts [2–4,14,15,17,31,32,34–37]. These cytokines play a prominent role in the immune-pathology of chikungunya arthritis by promoting inflammation, recruiting immune cells to the joints, and inducing the production of other inflammatory mediators. This cytokine storm contributes to the acute symptoms of chikungunya fever and arthralgia, as well as triggering events for subsequent chronic inflammation and joint damage.

#### Role of chemokines.

Chemokines are signalling proteins that recruit immune cells to sites of inflammation. In chikungunya arthritis, chemokines such as C-C motif ligand (CCL) 2, C-X-C motif chemokine ligand (CXCL) 9 and CXCL10 are upregulated in joints, leading to the infiltration of monocytes, macrophages, and T cells [3,15,17,38]. The immune cells involved contribute to the inflammatory response by producing additional cytokines and chemokines, creating a positive feedback loop that perpetuates inflammation. The persistent recruitment of immune cells to the joints, as well as viral persistence are key factors in the development of chronic arthritis.

#### Monocytes and macrophages.

These cells are believed to be the cellular reservoir and vehicle for dissemination and viral persistence [21]. Lum et al. highlighted the importance of macrophages in CHIKV through their experimental model using clodronate (an agent that depletes macrophages) in mice infected with CHIKV. These mice showed improved symptoms of CHIKV associated disease [31,39].

Elevated levels of monocyte chemotactic protein-1 (MCP-1) has been reported in numerous patient cohorts. The acute phase of the disease is associated with increased MCP-1 levels in patient serum [21,31,40–42].

Chow et al. emphasized the significance of granulocyte macrophage colony stimulating factor (GM-CSF), a pro-inflammatory cytokine. Higher serum levels occur during the chronic phase of the disease causing persistent arthralgia when compared to patients who had fully recovered in their case controlled longitudinal study [29].

#### Natural Killer (NK) cells.

NK cells are granular lymphocytes that form part of the innate immune system. NK cells are not a dominant population in synovial infiltrates they are present but less abundant than macrophages. CD69 + NK cells identified in synovial fluid (Hoarau et al.) may represent activated tissue-resident or infiltrating NK subsets, but causality in joint damage is unproven [21]. Their effect is predominantly local, however there is systemic involvement mediated via IFN-γ production and cytotoxic granules.

They also play a prominent role in immune-surveillance, cytotoxicity (through their release of cytotoxic granules), and cytokine production. These cells are present in vast numbers in inflamed joints following activation by IL-12 and IL-15 [14,21,32]. They aggravate the pathology through pro-inflammatory cytokine release, particularly IFN-γ production, which remain elevated in patients presenting with persistent arthralgia [3,31].

### 4. Cartilage and bone destruction

#### Synovitis and pannus formation.

**Fig 3:**
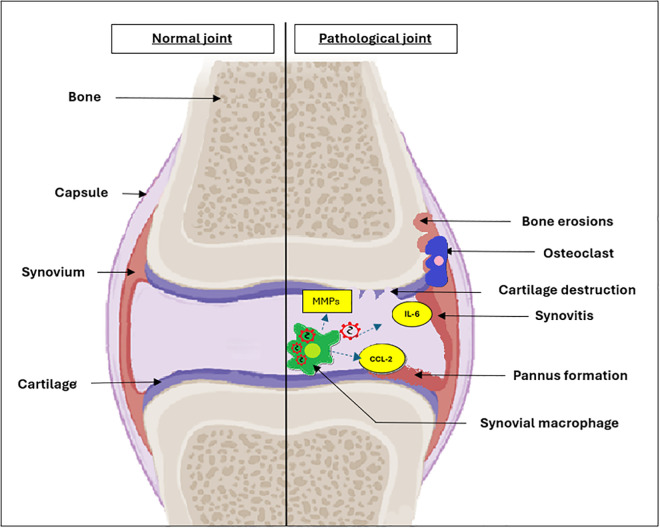
Illustration depicting the intraarticular pathological process. Infected macrophages within the synovium stimulate cytokine release which activates the destructive process of collagenolysis, osteolysis, synovitis and pannus formation.

Synovitis, or inflammation of the synovial membrane, is a hallmark of chikungunya arthritis [21]. The synovium becomes thickened and infiltrated with immune cells, including macrophages, T-cells, and B-cells [43]. This inflammatory infiltrate leads to the formation of pannus, a layer of granulation tissue that invades and erodes cartilage and bone (Fig 3) [44]. The pannus produces enzymes such as MMPs and cathepsins, which degrade the extracellular matrix of cartilage and bone, leading to joint destruction [19,23]. This pathological process closely resembles that seen in rheumatoid arthritis [2].

#### Role of matrix metalloproteinases (MMPs).

MMPs are a family of enzymes that degrade extracellular matrix components, including collagen and proteoglycans. In chikungunya arthritis, the expression of MMPs, particularly MMP-1 and MMP-3, is upregulated in serum and noted to be a biomarker of persistent arthralgia [32]. These enzymes are produced by fibroblast like synoviocytes and play a key role in the degradation of cartilage and bone, contributing to the structural damage observed in chronic chikungunya arthritis (Fig 3).

#### Osteoclast activation.

Osteoclasts are specialized cells that resorb bone tissue. In chikungunya arthritis, the activation of osteoclasts is mediated by the receptor activator of nuclear factor-kappa B ligand (RANKL), which is cytokines (IL-6, IL-1β) produced by synovial macrophages and activated T cells [45]. RANKL binds to its receptor RANK on osteoclast precursors, promoting their differentiation and activation. The increased activity of osteoclasts leads to bone resorption and erosion, contributing to the joint damage observed in chikungunya arthritis (Fig 3) [15,23,32,46].

#### Role of reactive oxygen species (ROS).

Reactive oxygen species (ROS) are highly reactive molecules that can damage cellular components, including DNA, proteins, and lipids. In chikungunya arthritis, the production of ROS is increased in synovial tissues, leading to oxidative stress and tissue damage [38]. Elevated oxidative stress markers have been observed in human cohorts. ROS amplify NF-κB (Nuclear Factor kappa-light-chain-enhancer of activated B cells) activation. This oxidative damage thus contributes to extracellular matrix degradation [19,23,38].

Additionally, ROS can induce the production of pro-inflammatory cytokines and chemokines, further exacerbating the inflammatory response in the joints [38].

#### Impact of cartilage and bone destruction on joint function.

The destruction of cartilage and bone in chikungunya arthritis has significant implications for joint function. Cartilage degradation leads to the loss of the smooth articular surface, resulting in joint stiffness, pain, and reduced mobility [23]. Bone erosion can lead to joint deformity and instability, further impairing joint function [35]. The structural damage to the joints is often irreversible, leading to long-term disability and reduced quality of life for patients with chronic chikungunya arthritis [47]. And it is these clinical features that gave rise to the name “Chikungunya” meaning “to be contorted” or “that which bends [2].

## Discussion

The chronic phase of CHIKV arthritis is said to mimic the clinical appearance of rheumatoid arthritis, and thus has been referred to in some texts as “Post Chikungunya chronic inflammatory rheumatism”, or even “seronegative spondyloarthritis” [2]. Both sets of conditions may presents with a symmetrical polyarthritis producing painful, swollen and stiff joints [2]. They are also characterised by raised serum inflammatory markers (i.e., CRP and ESR), with a similar serum cytokine profiles, barring the CCL-5 which is actually decreased in chronic phase of CHIKV arthritis, and elevated in RA [36]. However, in contrast, autoantibodies (i.e., anti-CCP and RF) are usually negative in Chikungunya arthritis, although an anti-CCP positivity rate of up to 28% was observed in one cohort [48].

Moreover, when comparing CA and RA it is noteworthy that they share several downstream immunological features. This includes the chronic synovitis and pannus formation. An elevated IL-6, TNF-α, and IFN-γ. An overexpression of MMP-1 and MMP-3. Osteoclast activation via the RANKL pathway, and persistent inflammatory infiltrates in the synovium [10].

However, key distinctions exist in the triggering mechanism whereby RA is an autoimmune disease with loss of tolerance and citrullinated antigen reactivity. This contrasts with CHIKV arthritis is which is triggered by post viraemia, with inflammation sustained by residual viral RNA proteins and innate immune activation.

Regarding the disease course, many CA patients partially improve over time, whereas RA typically requires continuous disease-modifying therapy. CA shows a stronger association with viral persistence markers and monocyte/macrophage activation signatures than with adaptive autoimmunity. Thus, although CA and RA appear similar clinically, their pathogenesis diverges substantially. This distinction supports the need for targeted therapies that address persistent innate inflammation rather than conventional RA management alone, though some immunomodulatory agents may still have a role to play [49,50].

Numerous synovial joint cell lines (i.e., FLS and synovial macrophages) that are noteworthy for discussion of CA that contribute to the immunobiology. Here we focus on the notable gaps within the literature relating to possible chondrocyte innate immune response.

To the best of our knowledge, none of the reviewed literature elaborated on the role of chondrocytes in the immune response, indicating a gap in our knowledge and a area for future study. Previous studies on the chondrocyte immune response have largely focused on the immunological facets of chondrocytes in osteoarthritis (OA), rather than in joint infection [51]. The literature that discusses OA suggests that chondrocytes do have an immunological function by way of receptor activation, cytokine secretion, and intra-cellular signalling. These roles are not only stimulated in pathological states, but also play a role in maintaining normal tissue homeostasis [51,52]. In OA, chondrocytes within the superficial layer of hyaline cartilage respond to cytokines when resident cells in joint surrounding tissues, such as macrophages and synoviocytes, secrete inflammatory cytokines into the synovial fluid. Those cytokines stimulate chondrocytes to produce pro-inflammatory mediators and extra cellular matrix (ECM) degrading enzymes. Both inflammatory mediators and ECM-degrading enzymes increase the severity of OA by promoting inflammatory progression and cartilage degeneration [19]. The inflammatory mediators secreted from chondrocytes also act in an autocrine fashion on chondrocyte stimulation. This leads to an increase in cartilage degrading enzymes (namely matrix metalloproteinases (MMPs) and a disintegrin and metalloproteinase with thrombospondin motifs (ADAMTS)) [19,23]. These mediators also activate inflammatory cytokine production, chondrocyte hypertrophy, chondrocyte maturation, chondrocyte apoptosis, and decrease of ECM production and chondrocyte proliferation [51,52].

Notably, this sort of detail describing the immune response of chondrocytes at a cellular level appeared to be absent during our search in the context of CHIKV.

Despite progress in understanding osteoarthritis and certain aspects of infectious arthritis, significant gaps remain in our knowledge of how chondrocytes contribute to immune defence within infected joints, particularly in the context of synovial macrophage interactions.

This review is not without limitations. As a scoping review, we did not quantitatively assess study quality or bias, and some included articles were narrative reviews themselves. There is a possibility we missed relevant literature, especially non-English publications and non-web based textbook literature. Additionally, many immunopathogenesis insights are drawn from cross-sectional studies or inferred from other related alphavirus models and in-vitro animal model tissue samples due to direct longitudinal data on CHIKV arthritis patients being limited.

The following research gaps can be noted to be given priorities within future studies.

Most current CHIKV arthritis data are derived from cross-sectional cohorts. Future studies should incorporate longitudinal sampling across acute and chronic phases of disease. Serial synovial biopsies or advanced joint imaging Integration of viral load measurements with immunophenotyping.

Only a few studies employ transcriptomics or proteomics. None have been known to use integrated multi-omics or single-cell technologies. Such approaches would greatly enhance understanding of cell-specific responses and persistence mechanisms.

Overall, there appears to be a lack of standardized protocols with variations in; sample collection, cytokine quantification, case definitions and arthralgia severity scoring.

This limits meaningful comparison across cohorts. Standardization is essential for building reproducible datasets.

Persistence mechanisms are noted to be poorly characterized. It remains unclear which viral components persist, whether persistence occurs uniformly or in focal niches and how persistent antigens interact with innate immunity.

Robust mechanistic studies using human-derived synovial cells and three-dimensional joint models are needed.

The therapeutic implications remain largely unexplored. Given the immunopathogenic similarities with RA, disease-modifying therapies may hold promise, but few trials have evaluated their use in CA.

## Conclusion

The pathogenesis of chikungunya arthritis is a complex and multifactorial process involving host factors, viral persistence, inflammatory triggers, and signalling pathways, as well as cartilage and bone destruction. Host factors such as genetic predisposition, age, sex, and comorbidities influence the susceptibility to, and severity of chikungunya arthritis. Viral persistence in joint tissues contributes to chronic inflammation and tissue damage, while inflammatory triggers and signalling pathways perpetuate the inflammatory response. The destruction of cartilage and bone is mediated by the activation of MMPs, osteoclasts, and ROS, leading to joint dysfunction and long-term disability in a disease process reminiscent of rheumatoid arthritis. This understanding provides a foundation for developing interventions by both orthopaedic practitioners and physicians alike to mitigate long-term disability from chikungunya arthritis.

## Supporting information

S1 Checklist PRISMA-ScR checklist. (PDF)

S1 Data Data screening tool. (XLSX)
